# Round the Hospitals

**Published:** 1916-06-10

**Authors:** 


### ROUND THE HOSPITALS.

We pointed out last week that the Council of the College of Nursing is busy formulating regulations. This fact takes but a few words to record, but it is to be wished that matrons, nurses, and hospital managers may realise the intricate difficulty and widespread importance of this work to the nursing profession as a whole. It is a grave and responsible task, and its successful accomplishment can only be secured by wise counsel evolved through discussion extending over an adequate period of time. Hurry in matters of this kind is fatal, and so, too, would be avoidable delays. It is far better for the Council to move with the maximum of caution at the outset than to allow itself to be unduly hastened to a decision less than the best.

AMONG the smaller matters which have an importance to nurses is the question of the registration fee. The Nurses Registration Bill gave power to the Registration Council to exact from every candidate for examination or registration fees not exceeding three guineas for examination and two guineas for registration. That is, a total initial fee of five guineas. We have always held, and hold still, that such fees are unjustifiable, and ought not to be demanded from nurses. In addition to the initial fee every registered nurse under the Bill has to pay 2s. 6d. every year, with a capital penalty of removal from the Register when in default, with restoration to it on a payment not exceeding 5s., when the failure has been due to inadvertence or mistake. The College of Nursing in its articles provides that a nurse who joins the Register may become a member of the College by a single payment of £1, with the option to pay in lieu thereof five annual instalments of 5s., that is 25s. in all. The Council will have to consider, seeing that membership of the College is dependent upon certification, whether or not to charge any registration fee in addition to the single payment of £1 for membership of the College. We hope that the Council may decide to admit qualified nurses to the Register and to make them members of the College for an inclusive fee of £1. If this is found possible it will be a striking proof of the fact that the Council intends the interests of the nurses to be its first consideration from the outset. Nurses have often a hard struggle to live, and the high fees rendered possible under the Registration Bill prove it to have been drafted without due regard to their circumstances and claims.

---

WHAT constitutes a training-school? is a question which has never so far been answered completely. Following the practice of the Medical Council, a hospital with 100 beds, with a training-school attached, has been roughly accepted as one which might be regarded as capable of training and granting certificates to nurses. There have been instances, as in the case of Taunton, which has nominally 109 beds, where the occupied beds have fallen on occasion to below fifty, and where during some months an even lower number of occupied beds may prevail. This is only a type, and a reference to successive volumes of "Burdett's Hospitals and Charities" will show each year in detail how many hospitals containing nominally 100 beds have on an average many fewer constantly occupied. It follows that a nominal bed-capacity cannot be regarded by the College or any recognised authority as a satisfactory basis for deciding whether a given institution can properly be recognised as a training-school for nurses. As the demand for nurses has increased in certain branches of the nursing field beyond the supply there has been a tendency to adopt as an index the average occupied beds as the standard, but when that standard is dropped to seventy-five occupied beds, as in the case of the Queen Victoria Jubilee Nurses' Institute, and to forty beds, as we are informed, in the case of the Royal British Nurses' Association, it will be seen that the difficulties and dangers which present themselves in arriving at a decision are neither slight nor unimportant. We are glad to learn that the Council of the latter body has been giving its attention to this question and has resolved to raise its standard of bed-capacity, eighty to one hundred beds being suggested.

---

WHAT the Council of the College of Nursing has to bear in mind is that it cannot recognise as a training-school any institution where the average number of in-patients, the character of the cases, and the arrangements made for technical and general training are not up to the modern standard. It requires to be plainly stated and clearly understood that a relatively small number of in-patients and the poor quality of the cases must have a material effect upon the training of nurses. For it is improper, if it is not even impossible, to take probationers for training at an institution where their number is in excess of the continuous facilities which can be provided for their practical instruction and training in the art of nursing. In any case, whatever its first decision, the Council of the College will be wise to proceed tentatively, so as to cause as little disturbance of existing arrangements as possible. The period of grace, another point awaiting the Council's decision, may prudently be used as a bridge between the ill-regulated arrangements of the present and the well-organised and adequate co-ordination of the whole of the available facilities for training nurses, in the future, which the hospitals of this country can be encouraged and helped to provide.

---

THE King's Birthday Honours announced on Saturday include the names of no fewer than 514 members of the various Nursing Services on whom the Royal Red Cross has been bestowed. Of these upwards of 200 are principal matrons, matrons, acting matrons, sisters, or acting sisters, members of Queen Alexandra's Imperial Military Nursing Service and its Reserve, Queen Alexandra's Military Nursing Services for Australia and India, the Nursing Services of the Territorial Force, the Australian Army, Canada, and New Zealand, the Civil Hospitals Reserve, the Order of St. John and British Red Cross Societies, the American Nursing Service, and the nursing staffs of military and war hospitals and of civil hospitals. Lady Perrott, Lady Superintendent-in-Chief of the St. John Ambulance Brigade Voluntary Aid Detachments, has also been awarded the Royal Red Cross, First Class. We offer our sincere congratulations to all these ladies on the high honour which has been conferred on them. Readers of THE HOSPITAL will be interested to note that the list of those to whom the R.R.C., First Class, has been awarded includes the name of Miss E. C. Barton, principal matron of the 3rd London General Hospital and matron of Chelsea Infirmary, the omission of which from a previous Honours List we criticised in our issue of January 22, 1916, page 357.

**For Matrons' and Sisters' Appointments see page vi.**

